# Placentation in dolphins from the Amazon River Basin: the Boto, Inia geoffrensis, and the Tucuxi, Sotalia fluviatilis

**DOI:** 10.1186/1477-7827-5-26

**Published:** 2007-06-28

**Authors:** Vera MF da Silva, Anthony M Carter, Carlos E Ambrosio, Ana F Carvalho, Marina Bonatelli, Marcelo C Lima, Maria Angelica Miglino

**Affiliations:** 1 National Institute of Amazonian Research, Manaus, AM, Brazil; 2 University of Southern Denmark, Odense, Denmark; 3 University of Sao Paulo, Sao Paulo, SP, Brazil; 4 School of Veterinary Medicine, Sao Joao da Boa Vista, SP, Brazil

## Abstract

A recent reassessment of the phylogenetic affinities of cetaceans makes it timely to compare their placentation with that of the artiodactyls. We studied the placentae of two sympatric species of dolphin from the Amazon River Basin, representing two distinct families. The umbilical cord branched to supply a bilobed allantoic sac. Small blood vessels and smooth muscle bundles were found within the stroma of the cord. Foci of squamous metaplasia occurred in the allanto-amnion and allantochorion. The interhemal membrane of the placenta was of the epitheliochorial type. Two different types of trophoblastic epithelium were seen. Most was of the simple columnar type and indented by fetal capillaries. However, there were also areolar regions with tall columnar trophoblast and these were more sparsely supplied with capillaries. The endometrium was well vascularised and richly supplied with actively secreting glands. These findings are consistent with the current view that Cetacea are nested within Artiodactyla as sister group to the hippopotamids.

## Background

Little is known of placentation in whales [1]. Turner [2] showed that they have epitheliochorial placentation. For the baleen whales (suborder Mysticeti) our knowledge has advanced little further [3], since available specimens often have been in too poor condition for histology [4]. There is an excellent study of the early development of the fetal membranes in the humpback whale [5], but it does not extend to the establishment of the placenta. Rather more is known about the toothed whales (suborder Odontoceti), although based on descriptions of single specimens of bottlenose dolphin [6], harbour porpoise [7,8], killer whale [9], right whale dolphin [1] and Commerson's dolphin [1]. A brief account of placentation in the Ganges river dolphin was based on examination of four pregnant females [10].

Several lines of evidence point to a close relationship between the mammalian orders Cetacea and Artiodactyla (even-toed hoofed mammals). The argument is supported by recent fossil evidence [11,12] as well as by molecular phylogenetics, which has Cetacea nested within Artiodactyla and closely related to hippopotami [13-18]. This new interpretation of the phylogenetic affinities of cetaceans makes it interesting to re-examine their placentation and ask what morphological transformations occurred in the lineage of these aquatic mammals.

The Amazon River Basin is unusual in harbouring two sympatric species of cetacean, the small gray dolphin or tucuxi (*Sotalia fluviatilis *[19]) and the pink dolphin or boto (*Inia geoffrensis *[20]). Features shared by *Inia *and *Sotalia *include a reproductive cycle determined by the annual flooding cycles of the Amazon. Calving takes place when water levels are low or declining and fish more concentrated and susceptible to predation [21]. It is thought that *Inia *entered South America in the Miocene, at which time drainage of the Amazon Basin was towards the Pacific [20]. Subsequent uprising of the Andes led to clockwise reversal of the drainage of the Amazon. There was also disruption of a much larger river system, resulting in the isolation of three populations of *Inia *in the Orinoco Basin, the Amazon Basin and, separated by a waterfall barrier, in the Amazon tributaries of eastern Bolivia [22]. *Sotalia *is thought to have entered the Amazon Basin more recently, from the Atlantic, becoming the first non-platanistoid dolphin to live exclusively in fresh water [23]. The speciation event that separated it from the coastal *S. guianensis *has been dated to the Pliocene [23] or early Pleistocene [24].

Information about reproduction in river dolphins is sparse [21]. From time to time, individuals that have drowned in fishermen's nets are brought to the National Institute of Amazonian Research in Manaus and preserved for different studies. We have examined the placentae of these dolphins, which represent two distinct families: *I. geoffrensis *is an Iniidae and *S*. *fluviatilis *is the only freshwater species of the marine dolphin family Delphinidae.

## Methods

Three specimens of *I. geoffrensis *and one of *S. fluviatilis *were available for study at the National Institute of Amazonian Research (Table 1). The uterus had been opened and a large portion, with the placenta in situ and the fetus attached by the cord, had been fixed in 10% formalin. An additional specimen of *I. geoffrensis *from the Museum of Anatomy, School of Veterinary Medicine, University of Sao Paulo, consisted of fetus and membranes detached from the uterus and fixed in formalin. After examining the gross morphology, pieces of tissue were excised and processed for histology by standard methods. The paraffin-embedded tissue was sectioned at 5 μm and stained with haematoxylin and eosin (H.E.), Masson's triple stain, or by the periodic acid Schiff reaction (PAS).

**Table 1:** Morphometric data

	Fetal weight (g)	Crown-rump length (cm)	Length of cord (cm)
*Inia geoffrensis *(MA-993)	1,900	50	-
*Inia geoffrensis *(Toc 16)	2,000	40	13
*Inia geoffrensis *(F34/A)	4,200	57	21
*Inia geoffrensis *(FMVZ)	1,500	43	21
*Sotalia fluviatilis *(F-05)	3,500	61	-

## Results

### Gross anatomy

Similar features were found for both species under discussion. The umbilical cord contained two arteries, two veins and the urachus. After about 20 cm, the cord divided into two branches each containing a single artery and vein. Pigmented tissue accretions of variable size and irregular distribution were found on the surface of the cord.

The amnion and allantois were translucent membranes and appeared to be avascular. The allantois was the thicker membrane and had a rugous surface. The two membranes were juxtaposed over an extensive area, forming an allanto-amnion. The surface of the allanto-amnion was covered with small accretions. In addition, accretions occurred within the allantochorion and near its surface.

The uterine surface was trabecular in appearance. In most places the placenta adhered closely to the uterine wall, but there were areas where it readily detached. We also found some areas of smooth chorionic membrane that were less well vascularised, but were unable to ascertain their extent.

### Umbilical cord

**Figure 1:**
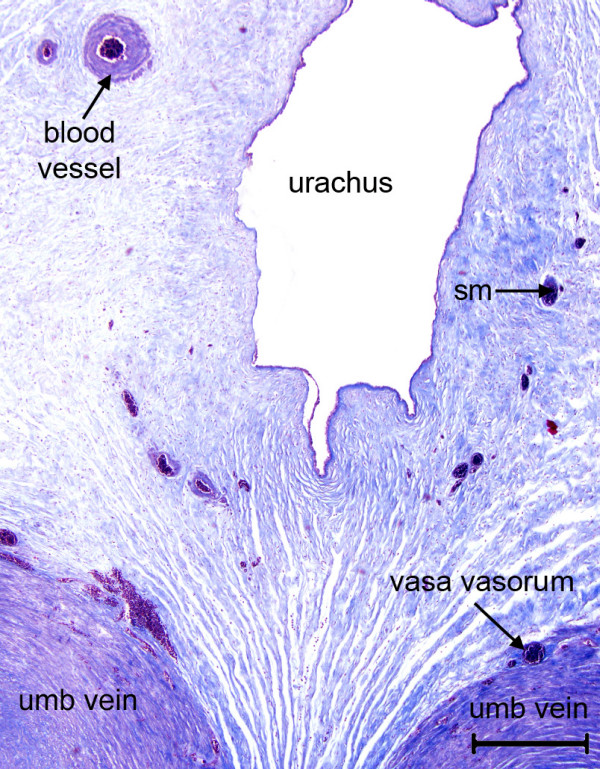
Umbilical cord of the tucuxi, *Sotalia fluviatilis*. Shown are part of the urachus and the paired umbilical veins with their vasa vasorum. The stroma is supplied with small blood vessels and contains bundles of smooth muscle (sm). Masson's triple stain. Scale bar = 500 μm.

**Figure 2:**
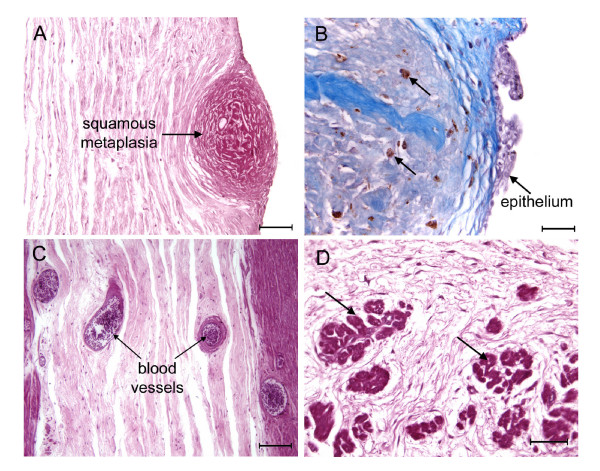
Umbilical cord of the boto, *Inia geoffrensis *(A, C-D) and the tucuxi, *Sotalia fluviatilis *(B). (A) Plaque of squamous metaplasia on surface of the cord. PAS. (B) Plaque of squamous metaplasia invested by stratified squamous epithelium. Note the presence of macrophages within the tissue (arrows). Masson's triple stain. (C) Blood vessels in the stroma of the cord. PAS. (D) Smooth muscle bundles (arrows) in the stroma of the cord. PAS. Scale bars = 100 μm (A, C), 50 μm (B, D).

The principal features of the cord are shown in Figure 1. Most of the surface was covered by simple squamous epithelium. The surface accretions were plaques of squamous metaplasia (Figure 2A). They were invested with stratified squamous epithelium (Figure 2B) and sometimes contained macrophages. The urachus was lined with transitional epithelium. The major umbilical vessels, veins as well as arteries, were endowed with vasa vasorum. The stroma of the cord was supplied with small blood vessels (Figure 2C). Smooth muscle bundles were seen within the stroma at some distance from the tunica media of the major vessels (Figure 2D) and running longitudinal to the cord.

### Fetal membranes

**Figure 3:**
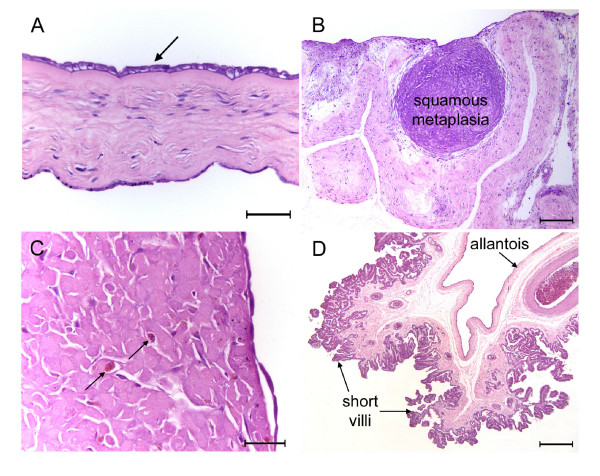
Fetal membranes of the tucuxi, *Sotalia fluviatilis *(A, C-D) and the boto, *Inia geoffrensis *(B). (A) The inner surface of the amnion (arrow) is lined by simple cuboidal epithelium. The outer mesothelium forms a simple squamous epithelium. H.E. (B) Folds of allanto-amnion with an area of extensive squamous metaplasia H.E. (C) Area of squamous metaplasia found close to the placenta showing macrophages (arrows) within the tissue. H.E. (D) Allantochorion from an area with poorly developed villous trees and short villi. H.E. Scale bars = 50 μm (A, C), 200 μm (B) 500 μm (D).

The amnion and allanto-amnion were avascular in both species. The inner surface of the amnion was covered by simple cuboidal epithelium, apparently with a brush border, and the outer surface with simple squamous epithelium (Figure 3A). Simple squamous epithelium also covered the surface of the allanto-amnion. There were large foci of squamous metaplasia at some places within the allanto-amnion (Figure 3B). They were also found near the chorioallantoic plate. They were invested with simple cuboidal or squamous epithelium and housed macrophages (Figure 3C).

### Uterus and placenta

**Figure 4:**
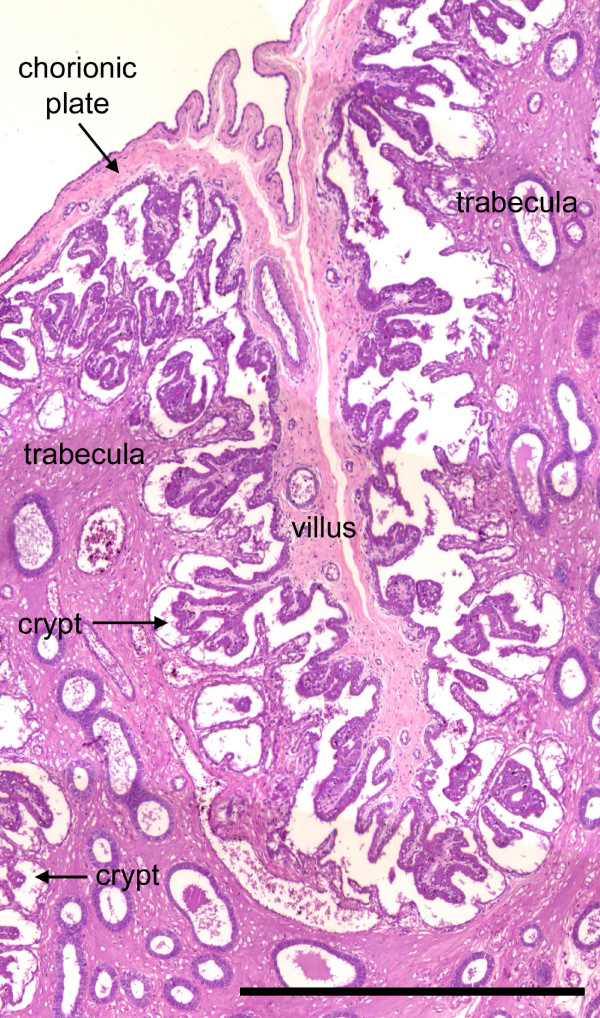
Placentation in the boto, *Inia geoffrensis*. The glandular endometrium forms trabeculae lined with uterine epithelium. Branched chorionic villi are inserted into the crypts. The spaces between the trophoblast and uterine epithelium are likely shrinkage artefacts. H.E. Scale bar = 1000 μm.

The interhemal membrane was of the epitheliochorial type. The uterine trabeculae interdigitated with folds of allantochorion that bore the fetal villi (Figure 4). Spaces occurred between the trophoblast and uterine epithelium. As discussed below, they likely were shrinkage artefacts.

**Figure 5:**
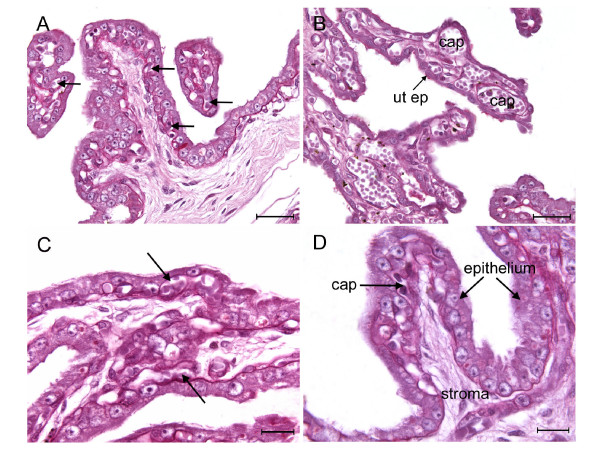
Placentation in the boto, *Inia geoffrensis*. (A) Maternal component of a placental exchange area showing uterine epithelium well supplied with capillaries (arrows). PAS. (B) The simple cuboidal uterine epithelium (ut ep) is closely underlain by the capillaries (cap). PAS. (C) Fetal villi from an exchange area showing the rich vascular supply and indentation of the trophoblast by capillaries (arrows). PAS. (D) Columnar trophoblastic epithelium with few capillaries (cap) supplied from the underlying stroma. PAS. Scale bars = 40 μm (A-B), 20 μm (C-D).

The endometrium showed no sign of decidualisation. It was well vascularised (Figure 5A) and richly supplied with glands lined by actively secreting simple columnar epithelium. The cuboidal uterine epithelium was partly indented by the maternal capillaries (Figure 5B).

The folds of allantochorion were invested with two different types of trophoblastic epithelium. Usually it was of the simple low columnar type indented by the capillaries with which it was so richly supplied (Figure 5C). We were also able to observe areolar regions where the trophoblast was of the tall columnar type with an apparent brush border (Figure 5D). These regions were more sparsely supplied with fetal capillaries. Columnar trophoblast also lined the stretches of chorionic plate between the villous trees (not shown). In some parts of the placenta the fetal villi were short and during fixation had lost contact with the maternal surface (Figure 3D).

## Discussion

The features of the cord, including its bifurcation to supply a bilobed allantoic sac, are similar to those described for other cetaceans [25]. The vascularisation of the stroma is discussed at some length by Wislocki [7]. Bundles of smooth muscle running longitudinally in the stroma of the cord were also shown in *Phocoena *[7]. The allanto-amnion was avascular in *Inia *and *Sotalia *as in *Tursiops *[6]. Small blood vessels were observed here in *Phocoena *[7]. Plaques of squamous metaplasia were found within and near the allanto-amnion and allantochorion of *Inia *and *Sotalia*, as has been described for other cetaceans [1].

Careful examination of the gravid uterus of *Tursiops *enabled Wislocki and Enders [6] to distinguish two regions of the placenta that were thicker, better vascularised and more closely adherent to the uterus. These were at the tubal end of the left horn, which housed the fetus, and in the right horn. Throughout most of the left horn the chorion was non-adherent and relatively avascular. As noted we did find some areas of smooth chorionic membrane with shorter villi and others that were less well vascularised, but were unable to ascertain their extent. In the blocks of tissue taken for histology, the branched chorionic villi and endometrial folds are interlocked, but spaces occur between the trophoblast and uterine epithelium. These appear in other studies [7,8] and it has been suggested that they are not just shrinkage artefacts but may represent natural spaces filled with embryotrophic material. In another odontocete, *Globicephala*, the trophoblast appears closely apposed to the uterine epithelium, except in regions that were more clearly adapted for embryotrophic nutrition. The latter are characterised by columnar trophoblast enclosing spaces that contain uterine gland secretion [26]. As mentioned by Wislocki [7], they tend to occur between the tips of the villi and the fundament of the crypts. In *Inia *and *Sotalia*, we found quite extensive regions of branching villi lined by columnar trophoblast. Columnar epithelium was also found immediately beneath the chorionic plate.

In their brief description of *Globicephala*, Morton and Mulholland [26] mention that the maternal capillaries in many crypt walls are intra-epithelial. This is borne out by our own observations on *Globicephala *at the Mossman Collection (University of Wisconsin Zoological Museum), but was not seen in either *Inia *or *Sotalia*, although the maternal capillaries did indent the epithelium. We agree with Wislocki and Enders [6] that intraepithelial capillaries do not occur on the fetal side as had been suggested by Ten Cate Hoedemaker [8].

In many odontocetes only the left ovary is active [27]. Consequently, the fetus is always found on the left side of the uterus, although the placenta occupies both horns [6]. Unfortunately, we did not receive information about the side of pregnancy in our specimens. This would have been of interest, since Best and da Silva [21], found scars in both ovaries of all four *Inia *that they examined. In the franciscana, *Pontoporia blainvillei*, which is a close relative of *Inia *[28,29], the right ovary was poorly developed [30]. In *Sotalia fluviatilis *ovarian scars were observed only in the left ovary [21] although both ovaries are active in the coastal species, *S. guianensis *[31].

### Phylogenetic implications

Until quite recently mammalian phylogenetics dealt with cladistic analysis of skeletal and dental characters. New insights concerning relations between the various orders have been provided by molecular tools, primarily analysis of coding sequences in mitochondrial and nuclear genes [13,15-18] often supplemented by molecular characters such as short and long interspersed elements [14,32] that are less subject to homoplasy. DNA sequence analysis consistently finds Cetacea nested within Artiodactyla. Therefore it is now common to consider cetaceans as part of a wider ordinal clade called Cetartiodactyla. Secondly, the whales and dolphins resolve as the sister group to hippopotamids. This contrasts with the traditional view that hippopotamids are more closely related to pigs and tayassuids. The new arrangement receives robust support from a meta-analysis for all living species of Cetartiodactyla that incorporates morphological as well as molecular data [33]. Recently it was shown that the foot bones of primitive Artiodactyla and early Cetacea exhibited shared, derived characters or synapomorphies [11]. Finally, renewed examination of the osteological characters of fossil and extant artiodactyls, including two early cetaceans, found that pigs and tayassuids formed a monophyletic group to the exclusion of hippopotamids, while cetaceans and hippopotamuses formed another monophyletic group [34].

These recent advances in phylogenetics have led to a re-evaluation of fetal and placental membrane characters [35-37]. Here we find that the placentas of *Sotalia *and *Inia *share a common pattern similar to that described for other cetaceans. It is apt to evaluate this pattern in relation to concepts such as the newly erected superorder Laurasiatheria, the combined order Cetartiodactyla and perceived relationships with the hippopotamids. Several characters in the placentation of Cetartiodactyla are shared with two other orders within Laurasiatheria, the horses and tapirs (Perissodactyla) and the scaly anteaters (Pholidota). These include the diffuse, villous and epitheliochorial placenta, and the presence of areolae [36,37]. Contrary to common perceptions, recent cladistic analyses agree in finding epitheliochorial placentation to be a derived state and link it to another shared character, the birth of well-developed (precocial) young following a relatively long period of gestation [35-40]. Indentation of trophoblast by fetal capillaries, and uterine epithelium by maternal ones, is common in these placentae and serves to reduce the diffusion distance [39]. Uterine gland secretions are an important source of nutrients in epitheliochorial placentae [35]. Columnar trophoblast, which is able to absorb these nutrients, is found in areolae and in the chorionic fossae [41]. Vascularisation of the cord stroma seems to be another common feature in Cetartiodactyla [7,25].

There are further resemblances in cetacean placentation to that of pigs, tayassuids and hippopotami. However, some characters are shared by hippopotami [42-44] and cetaceans, but not by pigs and tayassuids, in accordance with the current view that hippopotamids are the sister group to cetaceans. These features include a bilobed allantoic sac occupying both horns of the uterus, paired umbilical veins, and plaques of squamous metaplasia on the surface of the cord and amnion. It is interesting that the hippopotamus placenta, like that of *Tursiops *[6], has several distinct areas. One, with intimate interdigitation of chorionic villi and endometrial folds, is found mainly in the mesometrial region; the second is characterised by stunted villi and thinner endometrium; finally there is some relatively avascular chorion at the poles of the chorion and over the cervix.

In conclusion, placentation in *Inia *and *Sotalia *is similar to what has been described for other toothed whales. The limited variation in placental structures is consistent with the view that Odontoceti are monophyletic and derived from a small population of a common ancestral species [32]. Secondly, the epitheliochorial placenta resembles that of even-toed hoofed mammals [45] in accordance with the current view that whales are nested within the larger clade Cetartiodactyla [12]. Finally, some details of the placenta and fetal membranes support the view that whales are the sister group to hippopotamids [14].

## Authors' contributions

WMFS and MAM devised the study, participated in its design and coordination and helped to write the manuscript. AMC participated in the study design and coordination and wrote the manuscript. CEA participated in the coordination of the study and the histological analysis. AFC, MB and MCL performed a large part of the histological analysis. All authors read and approved the final manuscript.
